# Evaluation of Revascularization in Different Suzuki Stages of Ischemic Moyamoya Disease by Whole-Brain CT Perfusion

**DOI:** 10.3389/fneur.2021.683224

**Published:** 2021-07-23

**Authors:** Qingdong Han, Feirong Yao, Zhengyu Zhang, Yabo Huang

**Affiliations:** ^1^Department of Neurosurgery, The First Affiliated Hospital of Soochow University, Suzhou, China; ^2^Department of Radiology, The First Affiliated Hospital of Soochow University, Suzhou, China

**Keywords:** moyamoya disease, whole brain CT perfusion, revascularization, hemodynamics, collateral circulation

## Abstract

**Objective:** This study compared the clinical features and hemodynamic characteristics of patients in different Suzuki stages of ischemic moyamoya disease (iMMD) before and after treatment with extracranial-intracranial (EC-IC) bypass surgery combined with encephalo-duro-myo-synangiosis and whole-brain computed tomography perfusion (WB-CTP).

**Methods:** A total of 126 patients in different Suzuki stages (II, III, IV, and V) of iMMD who underwent bypass surgery from April 2013 to August 2020 were included in this retrospective study. MIStar automatic analysis of Whole brain CT perfusion imaging software (WB-CTP, Apollo Medical Imaging Technology, Melbourne, Australia) was used. The patients also underwent WB-CTP 1 day before and 1 week and 3 months after the surgery. The relationships between hemodynamic parameters in WB-CTP including delay time (DT) > 3 s, relative cerebral blood flow (rCBF) < 30%, mismatch and mismatch ratio, and clinical outcomes were evaluated for different Suzuki stages, with comparisons between early (II and III) and late (IV and V) stages.

**Results:** Combined bypass surgery was performed in 161 hemispheres of 126 patients with iMMD. Brain volume with DT > 3 s was decreased 1 week (51.5 ± 11.8 ml, *P* < 0.05) and 3 months (41.5 ± 10.7 ml, *P* < 0.05) after bypass compared to 1 day before bypass (104.7 ± 15.1 ml) in early-stage patients. In late-stage patients, the volume was increased 1 week after bypass compared to the preoperative value (154.3 ± 14.7 vs. 118.3 ± 19.1 ml, *P* < 0.05). Preoperative brain volume with rCBF < 30% was lower (9.8 ± 3.9 vs. 33.5 ± 11.0 ml) whereas preoperative mismatch ratio was higher (11.2 ± 2.8 vs. 3.6 ± 1.6) in early-stage as compared to late-stage patients (both *P* < 0.05). A higher modified Rankin scale score (0–1) was achieved by early-stage patients than by those in the late stage (93.8 vs. 80.4%, *P* < 0.05) at the 3-month follow-up.

**Conclusions:** WB-CTP is useful for assessing the effectiveness of combined bypass/revascularization in different Suzuki stages of iMMD. Patients in the early stage of disease with higher preoperative brain volume with DT > 3 s and mismatch ratio show greater improvements in hemodynamic parameters and fewer postoperative complications associated with hemodynamic disturbance following bypass than patients in the late stage. Preoperative mismatch ratio can serve as a marker for assessing the status of collateral circulation in different Suzuki stages of iMMD.

## Introduction

Moyamoya disease (MMD) can lead to severe ischemic stroke in adolescent and adult patients (1). Extracranial-intracranial (EC-IC) revascularization by superficial temporal artery-to-middle cerebral artery (STA-MCA) bypass combined with encephalo-duro-myo-synangiosis (EDAMS) is regarded as an effective treatment for complicated lesions (2–4). However, the consistency between pre- and postoperative hemodynamic characteristics in patients with iMMD in different Suzuki stages, and whether these parameters are related to postoperative complications, is not known.

Whole-brain computed tomography perfusion (WB-CTP) is a useful technique for screening patients with ischemic stroke for treatment with mechanical recanalization or intravenous thrombolysis within a narrow time window (5, 6). Although WB-CTP has been applied to MMD to assess the feasibility of revascularization, the relationship between Suzuki stage, WB-CTP features, and clinical outcomes has not been reported (7).

In the present study, we investigated the hemodynamic features in WB-CTP of ischemic (i) MMD patients in different Suzuki stages before and after EC-IC revascularization combined with EDAMS, and evaluated the relationship between hemodynamic parameters and clinical outcomes in different Suzuki stages.

## Methods

### Patients

In this retrospective study, we analyzed 161 hemispheres in 126 patients (61 males and 65 females) with iMMD in different Suzuki stages (II, III, IV, and V) who underwent digital subtraction angiography (DSA) and EC-IC revascularization by STA-MCA bypass combined with EDAMS at the First Affiliated Hospital of Soochow University (Suzhou, China) from April 2013 to August 2020. The surgical and imaging procedures were approved by the ethics committee of the First Affiliated Hospital of Soochow University. All patients provided written, informed consent.

### Evaluation of Postoperative Hemodynamics

**Figure 1:**
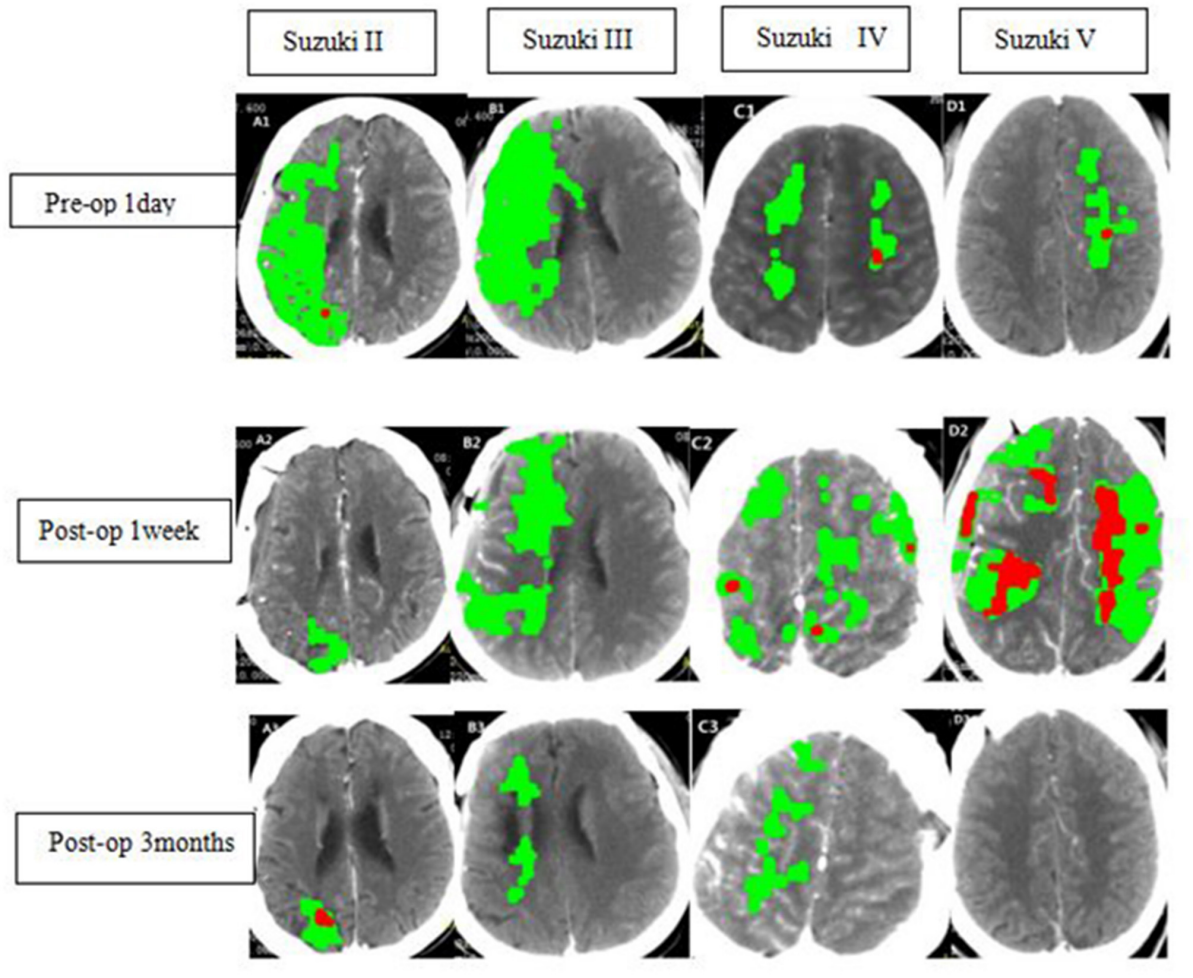
Radiological findings in ischemic MMD patients of different Suzuki stages at different time points. WB-CTP indicating the volume of ischemic penumbra (delayed time >3 s, green) and infarct core (relative cerebral blood flow <30%, red).A1-A3, B1-B3, C1-C3, D1-D3 showing WBC-CTP 1 day before bypass (Pre-op 1d), 1 week after bypass (Postop 1 week)and 3 months after bypass(Postop 3 months) of ischemic MMD patients at Suzuki stage II, III, IV, and V.

MIStar WB-CTP imaging software (Apollo Medical Imaging Technology, Melbourne, Australia) was used to evaluate pre- and postoperative hemodynamics as described in our previous study (8). WB-CTP was performed 1 day before and 1 week and 3 months after bypass. The measured hemodynamic parameters were delay time (DT) >3 s, relative cerebral blood flow (rCBF) <30%, and mismatch and mismatch ratios; these were compared between patients with Suzuki stage II, III, IV, and V disease and between early (II and III) and late (IV and V) stages (Figure 1). The mismatch was determined as brain volume with DT > 3 s minus brain volume rCBF; and mismatch ratio was determined as brain volume with DT > 3 divided by brain volume with rCBF <30%. rCBF was calculated as the absolute values of CBF in MCA terminal branches divided by the absolute values of CBF in cerebellar arteries. Two imaging experts blinded to the study analyzed the hemodynamic data.

### Treatment of Postoperative Complications

The following medical interventions were implemented to manage postoperative complications: antioxidant agents, hypotensive drugs, and dehydrating agents for cerebral hyperperfusion syndrome (CHS) (9); and antiplatelet agents for transient ischemic attack (TIA); and antiepileptic drugs and plasma concentration associated with drugs for epilepsy. Patients with minor hemorrhage without mass effect were treated with conservative treatment. The relationships between postoperative complications and hemodynamic parameters before and after bypass were analyzed.

### Follow-Up

Patients were followed up for a maximum of 36 months. Postoperative outcomes were assessed with the modified Rankin scale (mRS) score.

### Statistical Analysis

All variables are presented as mean ± standard deviations. Categorical variables were analyzed with the chi-squared test, and continuous variables were analyzed with the Student's or paired *t* test. Data analysis was performed using SPSS v23.0 (SPSS Inc, Chicago, IL, USA). Differences with *P* < 0.05 were considered significant.

## Results

### Demographic and Clinical Characteristics of the Study Population

The demographic and clinical characteristics of the study population are shown in Table 1. The mean age of the 126 patients was 46.3 ± 11.5 years (range, 15–78 years). The medical history of the cohort was as follows: hypertension, *n* = 29 (23.0%); diabetes mellitus, *n* = 24 (19.0%); elevated lipids, *n* = 22 (17.5%); smoking, *n* = 25 (19.8%); and alcohol abuse, *n* = 14 (11.1%). The number patients in each Suzuki stage was as follows: II, *n* = 11; III, *n* = 46; IV, *n* = 59; and V, *n* = 10. Preoperative clinical diagnosis included TIA (*n* = 75, 59.5%) and reversible ischemic neurologic deficit (RIND) (*n* = 51, 40.5%). A total of 161 hemispheres in the 126 patients were successfully treated by combined bypass/EDAMS. Postoperative complications included CHS (*n* = 16, 12.7%), TIA (*n* = 11, 8.7%), epilepsy (*n* = 10, 7.9%), and intracerebral hemorrhage (ICH) (*n* = 5, 4.0%).

**Table 1:** Baseline characteristics of 126 ischemic MMD patients.

**Suzuki stage**	**II(11)**	**III(69)**	**IV(36)**	**V(10)**
**Gender**				
Male (61)	4	30	21	6
Female (65)	7	39	15	4
**Age**				
>60 years (14)	1	7	5	1
≤ 60 years (112)	10	62	31	9
**Ischemic symptom**				
TIA (75)	7	38	22	8
RIND (51)	4	31	14	2
**Medical history**				
Hypertension(29)	2	15	9	3
Smoking (25)	4	10	9	2
DM (24)	1	13	8	2
High lipid (22)	2	11	7	2
Alcohol abusing (14)	1	7	9	2
**Pre-operative mRS Scale**				
0–1 (86)	9	47	23	7
2–3 (40)	2	22	13	3
**Revascularization side**				
Left side (50)	6	29	12	3
Right side (51)	3	26	16	6
Left and Right sides(25)	2	14	8	1
**Post-operative complication**				
CHS (16)	1	6	7	2
TIA (11)	1	5	4	1
Epilepsy(10)	0	2	2	1
Intra-cerebral hemorrhage (5)	0	2	2	1
**Post-operative mRS scale**				
0–1 (112)	10	65	28	9
2–3 (14)	1	4	8	1

*TIA, Transient ischemic attack; RIND, Reversible ischemic neurological deficit.*

*DM, Diabetes mellitus; CHS, Cerebral hyperperfusion syndrome; mRS, Modified Rankin Scale*.

### Hemodynamic Characteristics in Different Suzuki Stages of iMMD Before and After Bypass

#### Hemodynamic Characteristics in Different Suzuki Stages Before and After Bypass

**Figure 2:**
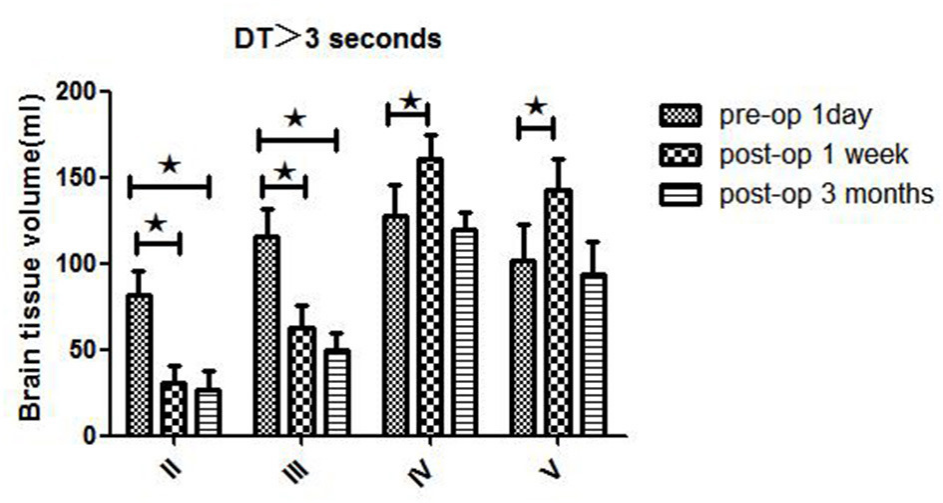
WB-CTP showing hemodynamic characteristics in DT > 3 s in ischemic MMD patients of different Suzuki stages (II–V stage) at different time points, ^⋆^*P* < 0.05.

Patients in different Suzuki stages showed different hemodynamic features in WB-CTP. Postoperative brain volume with DT > 3 s was significantly decreased 1 week (30.3 ± 10.2 ml) and 3 months (26.5 ± 11.5 ml) after bypass compared to the preoperative volume (81.7 ± 13.6 ml) (both *P* < 0.05) in stage II patients (Figure 2). The same trend was observed for patients with stage III disease (1 day before surgery: 115.6 ± 16.5 ml; 1 week after surgery: 62.4 ± 13.5 ml; 3 months after surgery: 49.5 ± 10.1 ml) (both *P* < 0.05). However, in stage IV patients, brain volume with DT > 3 s was increased at 1 week after bypass (160.5 ± 14.1 ml) compared to before the surgery (127.6 ± 17.7 ml) (*P* < 0.05), although no difference was observed at 3 months (119.7 ± 9.8 ml). The same was true for patients with stage V disease (1 day before surgery: 101.3 ± 21.4 ml; 1 week after surgery: 142.2 ± 18.6 ml; 3 months after surgery: 93.6 ± 19.2 ml).

**Figure 3:**
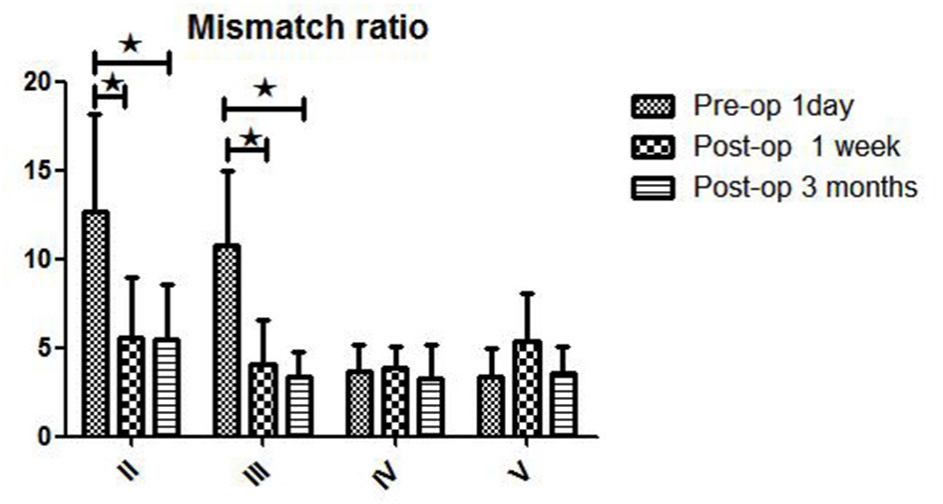
WB-CTP showing hemodynamic characteristics in DT > 3 s in ischemic MMD patients of different Suzuki stages (early and later stage) at different time points. Early stage: in Suzuki stage II and III Later stage:in Suzuki stage IV and V. ^⋆^*P* < 0.05.

The postoperative mismatch ratios 1 week (5.6 ± 3.4) and 3 months (5.5 ± 3.1) after bypass were significantly lower than the preoperative ratio (12.7 ± 5.5, *P* < 0.05) in stage II patients (both *P* < 0.05) (Figure 3 and Table 2). For patients in Suzuki stage III, a significant decrease compared to the preoperative mismatch ratio (10.8 ± 4.2) was observed only at the 1-week follow-up (4.1 ± 2.5) (*P* < 0.05), with no difference at 3 months (3.4 ± 1.4). Patients in stages IV and V showed no differences between preoperative and 1-week or 3-month postoperative mismatch ratios (stage IV: 3.7 ± 1.5 vs. 3.9 ± 1.2 vs. 3.3 ± 1.9; stage V: 3.4 ± 1.6 vs. 5.4 ± 2.7 vs. 3.6 ± 1.5) (both *P* > 0.05).

**Table 2:** WB-CTP showing hemodynamic characteristics in ischemic MMD patients of different Suzuki stages at different time points (1 day before bypass, 1 week after bypass and 3 months following bypass).

	**Pre-operative 1day**			**Post-operative 1 week**			**Post-operative 3months**		
	**DT>3s (ml)**	**rCBF <30% (ml)**	^*^MR (ml)	**DT>3s (ml)**	**rCBF <30% (ml)**	^*^MR (ml)	**DT>3s (ml)**	**rCBF <30% (ml)**	^*^MR (ml)
II	81.7 ± 13.6	7.0 ± 4.1	12.7 ± 5.5	30.3 ± 10.2	6.1 ± 2.6	5.6 ± 3.4	26.5 ± 11.5	5.4 ± 3.3	5.5 ± 3.1
III	115.6 ± 16.5	11.2 ± 3.5	10.8 ± 4.2	62.4 ± 13.5	15.8 ± 5.7	4.1 ± 2.5	49.5 ± 10.1	14.5 ± 6.7	3.4 ± 1.4
IV	127.6 ± 17.7	34.9 ± 11	3.7 ± 1.5	160.5 ± 14.1	41.2 ± 0.9	3.9 ± 1.2	119.7 ± 9.8	36.7 ± 13.7	3.3 ± 1.9
V	101.3 ± 21.4	31.7 ± 10.5	3.4 ± 1.6	142.2 ± 18.6	26.4 ± 1.7	5.4 ± 2.7	93.6 ± 19.2	25.5 ± 9.7	3.6 ± 1.5

* *MR, mismatch ratio (volume of DT > 3 divide volume of rCBF <30%)*.

#### Hemodynamic Characteristics in Early vs. Late Suzuki Stage Before and After Bypass

**Figure 4:**
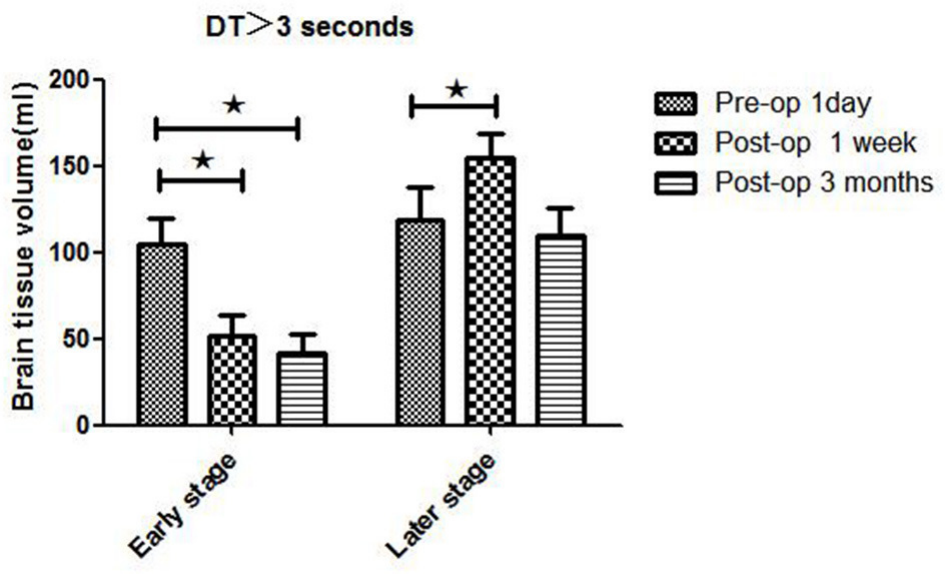
WB-CTP showing hemodynamic characteristics in mismatch ratio in ischemic MMD patients of different Suzuki stages (II–V stage) at different time points. Early stage: in Suzuki stage II and III Later stage:in Suzuki stage IV and V. *MR, mismatch ratio (volume of DT > 3 divide volume of rCBF < 30%), ^⋆^*P* < 0.05.

**Figure 5:**
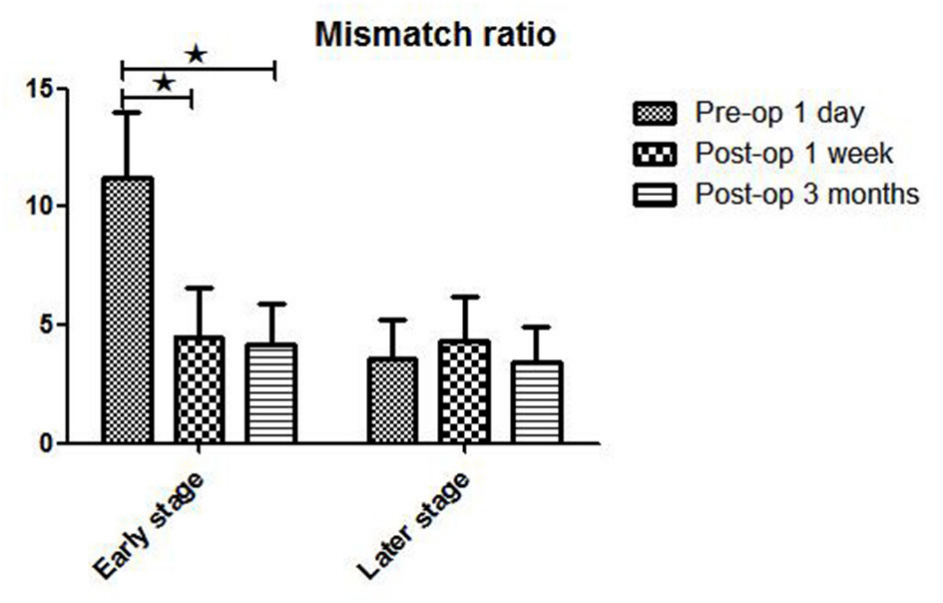
WB-CTP showing hemodynamic characteristics in mismatch ratio in ischemic MMD patients of different Suzuki stages (early and later stage) at different time points. Early stage: in Suzuki stage II and III Later stage:in Suzuki stage IV and V. *MR, mismatch ratio (volume of DT > 3 divide volume of rCBF < 30%), ^⋆^*P* < 0.05.

Hemodynamics characteristics of early-stage (II and III) and late-stage (IV and V) iMMD patients differed significantly. Postoperative brain volume with DT > 3 s was markedly lower 1 week (51.5 ± 11.8 ml) and 3 months (41.5 ± 10.7 ml) after bypass compared to the preoperative volume (104.7 ± 15.1 ml) (both *P* < 0.05) in early-stage patients (Figure 4 and Table 3). In contrast, in late-stage patients the volume was significantly higher 1 week after as compared to 1 day before the surgery (154.3 ± 14.7 vs. 118.3 ± 19.1 ml; *P* < 0.05), whereas no significant difference relative to the preoperative value was observed at the 3-month follow-up (109.5 ± 16.3 ml; *P* > 0.05). Early- and late-stage patients showed significant differences in mismatch ratio 1 day before bypass (11.2 ± 2.8 vs. 3.6 ± 1.6, *P* < 0.05), but no significant differences at 1 week (4.5 ± 2.1 vs. 4.3 ± 1.9, *P* > 0.05) and 3 months (4.2 ± 1.7 vs. 3.4 ± 1.5, *P* > 0.05) after bypass (Figure 5).

**Table 3:** WB-CTP showing hemodynamic characteristics in ischemic MMD patients of different Suzuki stages (in early stage and later stage) at different time points.

	**Pre-operative 1day**			**Post-operative 1 week**			**Post-operative 3months**		
	**DT>3s (ml)**	**rCBF <30% (ml)**	^*^MR (ml)	**DT>3s (ml)**	**rCBF <30% (ml)**	^*^MR (ml)	**DT>3s (ml)**	**rCBF <30% (ml)**	^*^MR (ml)
Early stage	104.7 ± 15.1	9.8 ± 3.9	11.2 ± 2.8	51.5 ± 11.8	11.6 ± 4.5	4.5 ± 2.1	41.5 ± 10.7	11.8 ± 5.5	4.2 ± 1.7
Later stage	118.3 ± 19.1	33.5 ± 11.0	3.6 ± 1.6	154.3 ± 14.7	35.7 ± 11.2	4.3 ± 1.9	3.4 ± 1.5	109.5 ± 16.3	32.4 ± 11.1

*Early stage: in Suzuki stage II and III Later stage: in Suzuki stage IV and V.*

* *MR, mismatch ratio (volume of DT > 3 divide volume of rCBF <30%)*.

### Relationship Between Hemodynamic Findings and Postoperative Complications in Different Suzuki Stages in iMMD

The rates of postoperative complications in different Suzuki stages were as follows: stage II, 2/11 (18.2%); stage III, 17/69 (24.6%); stage IV, 18/36 (50%); stage V, 5/10 (50%) (Table 1). The 4 major types of postoperative complication occurred at higher rates in late-stage as compared to early-stage patients, including CHS (19.6 vs. 8.6%), TIA (10.9 vs. 7.5%), epilepsy (13.0 vs. 5.0%), and ICH (6.5 vs. 2.5%).

Preoperative brain volume with rCBF <30% was lower in patients in the early stage (9.8 ± 3.9 ml) than in patients in the late stage (33.5 ± 11.0 ml) of iMMD (*P* < 0.05) (Table 3). Accordingly, preoperative mismatch ratio was higher in the early stage as compared to the late stage of disease (11.2 ± 2.8 vs. 3.6 ± 1.6; *P* < 0.05). Of the 80 patients in the early stage, 56 (70.0%) had a preoperative mRS score of 0–1; the number of patients with scores in this range increased to 75 (93.8%) at 3 months after surgery. Among the 46 patients in the late stage, 30 (65.2%) had an mRS score of 0–1 before the surgery and the number increased to 37 (80.4%) at 3 months after bypass; the difference between early- and late-stage patients at the 3-month follow-up was statistically significant (*P* < 0.05).

## Discussion

MMD is a type of cerebrovascular lesion whose etiology is not fully understood. Given the occlusion of intracranial arteries, establishing collateral circulation through EC-IC bypass can increase cerebral blood flow, thereby preventing ischemic stroke or hemorrhage (10–13). However, in order to ensure good clinical outcomes with this approach, it is important to understand the hemodynamic characteristics of iMMD patients in different Suzuki stages and postoperative complications associated with hemodynamic variances.

The iMMD patients in different Suzuki stages in our study showed differences in hemodynamic features in WB-CTP. A higher mismatch ratio and lower brain volume with rCBF <30% are known to be related to better collateral circulation, which has been used to guide the treatment of embolism in ischemic stroke (14). In the present study, the hemodynamic parameters of early Suzuki stage (II and III) patients markedly improved after bypass surgery. However, the same was not true of patients in the late stages (IV and V) of disease, who showed increased volumes of both the ischemic core and penumbra. The preoperative mismatch ratio was also lower in late-stage MMD patients, indicating an insufficiency of collateral vessels. Thus, preoperative mismatch ratio can be considered as a marker for the status of collateral circulation in different Suzuki stages of iMMD. Additionally, the possibility of a postoperative decrease in blood vessel formation following EC-IC revascularization and cerebral cortex perfusion should be noted (7). In our study, the postoperative mismatch ratio was decreased in both early- and late-stage patients following bypass, which is in accordance with previous reports (7).

The observed changes in collateral circulation and hemodynamics following bypass surgery were related to postoperative complications. Mismatch in the cerebral cortex is thought to reflect a salvageable brain area after ischemic stroke (5). EC-IC bypass can have a marked effect on collateral circulation, inducing hemodynamic disturbances such as cerebral hyperperfusion syndome and postoperative watershed shift ischemia (15). It has been reported that micro embolism was related to MMD progression and infarct onset (10). One study proposed that collateral circulation in MMD be classified based on whether the origin is the external or internal carotid or vertebral artery, as patients with collaterals originating from the external carotid artery usually had late-stage disease and were at a higher risk of infarction (16). So we protect trans-dural anastomosis, such as middle meningeal arteries, during craniotomy especially in patients with late Suzuki's stage. Previous report indicated the significant association of the risk of cerebral hyperperfusion with the higher Suzuki's angiographic stage (17).

Our study showed that the rate and severity of postoperative complications (CHS, TIA, epilepsy, and ICH) in early- vs. late-stage iMMD patients differed significantly. CTP provides a method for quantitatively evaluating cerebral perfusion; a discrepancy between cerebral blood flow and ischemic presentation indicates that both cerebrovascular reserve and collateral circulation are responsible for hemodynamic disturbances (18). In our study, the infarct core (rCBF <30%) and mismatch ratio differed markedly between early- and late-stage patients, implying poor collateral circulation and impaired auto-regulatory function of the cerebral vasculature in the latter group. Dramatic and sudden hemodynamic changes following bypass can result in excessive blood flow, leading to CHS or ICH (19). Postoperative watershed ischemia has been attributed to competition between high blood flow from the STA and intrinsically low or retrograde flow in the MCA (20).

Other imaging findings can facilitate the assessment of hemodynamic changes during EC-IC bypass in the treatment of iMMD. CO_2_-triggered blood oxygen level-dependent functional magnetic resonance imaging (MRI) can reveal regional cerebrovascular reserve or reactivity in MMD patients (21), but variations in breath holding across patients can influence the results. As a non-invasive tool, duplex ultrasonography has been used to examine collateral circulation pre- and postoperatively, allowing neurosurgeons to predict clinical outcomes (22, 23), although this may be susceptible to inter-observer variations. Image-based computational fluid dynamics in CTP can provide information on graft blood flow, but the distal portion of the STA graft is difficult to visualize, especially in pediatric patients (24). The ivy sign in the ipsilateral hemisphere in fluid-attenuated inversion recovery MRI before and after revascularization was shown to be an indicator of hyperperfusion (25). Finally, quantitative single-photon emission computed tomography (SPECT) can be used to monitor postoperative hemodynamic disturbances (20).

There were some limitations to our study that warrant attention. Firstly, we analyzed data from a single center, which could limit the generalizability of our findings. Secondly, only patients with stage II–V iMMD were included in the analysis; additional studies are needed to determine the hemodynamic profiles of patients in other stages (i.e., I and VI). Thirdly, our analyses were based on radiologic findings but data obtained from other imaging modalities and examinations should be considered in the evaluation of postoperative hemodynamic status.

## Conclusion

WB-CTP is a useful method for evaluating the effectiveness of combined revascularization in the treatment of iMMD. Patients in the early stages of disease (Suzuki stages II and III) can achieve greater improvements in hemodynamic parameters with fewer hemodynamic complications following EC-IC bypass surgery than those in the late stages (Suzuki stages IV and V). Preoperative mismatch ratio can serve as a marker for assessing the status of collateral circulation in different Suzuki stages of iMMD.

## Data Availability Statement

The raw data supporting the conclusions of this article will be made available by the authors, without undue reservation.

## Ethics Statement

The studies involving human participants were reviewed and approved by the Ethics Committee of the First Hospital of Soochow University. Written informed consent to participate in this study was provided by the participants' legal guardian/next of kin.

## Author Contributions

YH and QH: design of the study, writing—review and editing, and supervision. FY and QH: data resources. FY and ZZ: imaging analysis. QH and YH: writing—original draft. QH and FY: statistical analysis. All authors contributed to the article and approved the submitted version.

## Conflict of Interest

The authors declare that the research was conducted in the absence of any commercial or financial relationships that could be construed as a potential conflict of interest.

## Publisher's Note

All claims expressed in this article are solely those of the authors and do not necessarily represent those of their affiliated organizations, or those of the publisher, the editors and the reviewers. Any product that may be evaluated in this article, or claim that may be made by its manufacturer, is not guaranteed or endorsed by the publisher.

## Abbreviations

MMD: moyamoya disease
WB-CTP: whole brain CT perfusion
STA: superficial temporal artery
MCA: middle cerebral artery
DT: Delay time
rCBF: relative cerebral blood flow
CTA: computed tomography angiography
CTP: computed tomography perfusion
DSA: digital subtraction angiography
CHS: cerebral hyperperfusion syndrome
TIA: Transient ischemic attack
MRI: magnetic resonance imaging
mRS: modified Rankin scale score.
